# Supplementary material for: 3D covalent organic framework membrane with fast and selective ion transport
Source: Nat Commun. 2023 Sep 22;14:5926. doi: 10.1038/s41467-023-41555-5 (PMC10517170; doi:10.1038/s41467-023-41555-5)
Supplement: Supplementary file 1 — Supplementary Information [file 41467_2023_41555_MOESM1_ESM.pdf]

Supplementary Information for

3D Covalent Organic Framework Membrane with Fast and Selective Ion Transport

Tianhao Zhu[1,2‡], Yan Kong[1,2‡], Bohui Lyu[3], Li Cao[1,2], Benbing Shi[1,2], Xiaoyao Wang[1,2], Xiao Pang[1,2], Chunyang Fan[1,2], Chao Yang[1,2], Hong Wu[1,2], and Zhongyi Jiang[1,2,3*]

1. Key Laboratory for Green Chemical Technology of Ministry of Education, School of Chemical Engineering and Technology, Tianjin University, Tianjin 300072, China.

2. Haihe Laboratory of Sustainable Chemical Transformations, Tianjin 300072 (China).

3. Joint School of National University of Singapore and Tianjin University, International Campus of Tianjin University, Binhai New City, Fuzhou, 350207, China.

‡These authors contributed equally.

*Correspondence to: zhyjiang@tju.edu.cn (Z.J.)

This file includes:

Supplementary Fig. 1 to 28

Supplementary Table 1 to 4

References (1−45)

1. Materials and Experiments

Materials.

Tetra-(4-anilyl)-methane and 2,5-dihydroxyterephthalaldehyde were purchased from ET Co. Ltd (Jilin, China). Acetic acid, trifluoroacetic acid, p-toluene sulfonic acid, sodium carbonate and 1,3-propane sultone were purchased from Sigma-Aldrich Co. Ltd (Shanghai, China). Anhydrous octanoic acid, hexanoic acid, nonanoic acid, acetonitrile, hydrochloric acid, ethanol, mesitylene and tetrahydrofuran were purchased from Meryer Chemical Reagent Co. Ltd (Shanghai, China). Water used in all experiments was prepared by a Milli-Q ultrapure unit.

Synthesis of 3,3'-((2,5-diformyl-1,4-phenylene) bis(oxy)) bis (propane-1-sulfonic acid) monomer (monomer A).

Monomer A was synthesized according to a previous report of a similar compound with modified procedures[1]. 0.34 g (2 mmol) 2,5-dimethoxyterephthalaldehyde and 0.85 g sodium carbonate (8 mmol) were dissloved in 50 mL anhydrous acetonitrile, to which 1,3-propane sultone were added. The mixture was allowed to react at 80 °C for 10 h under N_2 protection. After cooling to room temperature, the crude product was collected by filtration and washed and purified by a reverse phase chromatography.

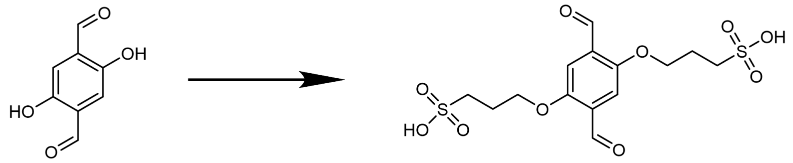

IEC values of 3D SCOF membrane.

The ion exchange capacity (IEC) values of the 3D SCOF membrane were measured by a titration method. The dry samples (0.2 g) were immersed in NaCl solution (0.01 g mL^{-1}, 20 mL) and stirred for 24 h. Then the solutions were titrated using a 0.01 M NaOH standard solution, and phenolphthalein was used as indicator. The IEC values (mmol g^{-1}) of the samples were calculated by the equation (1):

$$\mathrm{IEC} = \frac{C_{\mathrm{NaOH}} \times V_{\mathrm{NaOH}}}{W_{\mathrm{Dry}}} \quad (1)$$

where C_{NaOH} (mol L^{-1}) is the molar concentration of the NaOH solution; V_{NaOH} (L) is the consumed volume of the NaOH solution during the titration process; and W_{Dry} (g) is the weight of the dry samples.

Pore size distribution tests

The rejection tests of 3D SCOF membranes were conducted on a homemade dead-end filtration device at 2 bar based on polyethylene glycols (PEG) with various molecular weights (600, 1500, 2000, and 4000 Da). Before the rejection test, each PEG was dissolved in DI water to form a feed solution with a concentration of 200 ppm. Concentrations of PEGs in the feed and permeate solution were detected by total organic carbon (TOC) analyzer. The rejection rates (R) were calculated by the equation (2):

$$R = \left(1 - \frac{c_{\mathrm{f}}}{c_{\mathrm{p}}}\right) \times 100\% \quad (2)$$

where c_f and c_p are the solution concentration in the feed and permeate solution, respectively.

The average pore size was defined as same as the geometric mean diameter of the PEG with 50% rejection. The Stokes radii of PEG can be calculated from the equation (3):

$$r = 16.73 \times 10^{-12} \times Mw^{0.557} \quad (3)$$

Proton conductivity measurement.

The proton conductivity of the 3D SCOF membranes were measured using two-electrode alternating current (AC) impedance spectroscopy using an electrochemical workstation (Compact Stat, IVIUM Tech.). The membrane was cut into strip sample of 0.3-0.5 cm wide and clamped across two silver electrodes spaced 1 cm apart. Resistance value (Ω) was tested over the frequency in the range of 1000000^{-1} Hz with an oscillating voltage of 10 mV. Proton conductivity measurements under different relative humidity and temperature conditions were carried out in a thermo-hygrostat. For the long-term stability test, the membrane was

clamped between the two electrodes and stayed in the testing conditions (60°C, 100% RH) all the time. The proton conductivity (σ, mS cm^{-1}) of membranes were calculated by equation (4):

$$\sigma = \frac{l}{R \times A} \times 1000 \qquad (4)$$

Where l (cm), R (Ω), and A (cm^2) represented the distance between electrodes, resistance, and cross-sectional area of the samples, respectively. The E_a (kJ mol^{-1}) represents the activation energy for ion conduction can be defined by the following equation (5):

$$\ln \sigma = -E_a/(RT) + \ln \sigma_0 \qquad (5)$$

Osmotic energy conversion.

The 3D SCOF membrane was mounted in between a two-chamber cell wherein the chambers contained electrolyte solutions with different concentration. A pair of fresh Ag/AgCl electrodes (5 mm × 20 mm × 0.2 mm) were placed in the two side chambers. For the osmotic power output, the cell was connected to an external load resister (MC-21-B, Mingcheng, Shenzhen, China) and the current across the resistance was recorded using a source meter (KEITHLEY, 2450 SourceMeter®). The 3D SCOF membrane was fixed at a silicon holder with a central hole (diameter: ~ 200 μm). The effective area of membrane was 0.03 mm^2. For the long-term stability test, the membrane was immersed in the testing solution all the time, and the testing solutions were refreshed before each measurement.

The output power (P_L) and output power density (PD) were calculated using the following equations:

$$P_L = I^2 \times R_L \qquad (6)$$

$$PD = P_L/S \qquad (7)$$

where I is the current, R_L is the external load resistance, and S is the effective testing area of the membrane.

Energy conversion efficiency.

For diffusion potential (E_{diff}) of 3D COF membranes in asymmetric electrolytes (0.01M/0.5M NaCl solution), a salt bridge was used to eliminate the redox potential from Ag/AgCl electrodes (commercial Ag/AgCl wire electrodes, diameter: ~ 0.5 mm, charged with salt bridges).

E_{diff} can be calculated as: $E_{diff} = V_{oc} - E_{redox}$, where V_{oc} and E_{redox} refer to the open-circuit voltage and redox potential, respectively. The generation of E_{redox} is avoided by connecting the electrodes to the cell with agarose salt bridges. Therefore, the E_{diff} value is directly obtained from the intercept on the voltage axe of the current-voltage (I–V) curves.

For a given concentration gradient, the cation transference number (t) can be calculated as:

$$t = \frac{1}{2}\left(\frac{E_{diff}}{\frac{RT}{zF} ln\left(\frac{\gamma_{CH} C_{H}}{\gamma_{CL} C_{L}}\right)} + 1\right) \quad (8)$$

where R, T, F, z, γ and c refer to the universal gas constant, absolute temperature, Faraday constant, charge number, activity coefficient of ions, and ion concentration, respectively.

Accordingly, the maximum energy conversion efficiency (η_{max}) can be calculated as:

$$\eta_{max} = \frac{1}{2}(2t - 1)^2 \quad (9)$$

When the membrane is ideally cation selective, t reaches the maximum value of 1, and the maximum efficiency reaches the maximum value of 50%."

2. Figures and tables

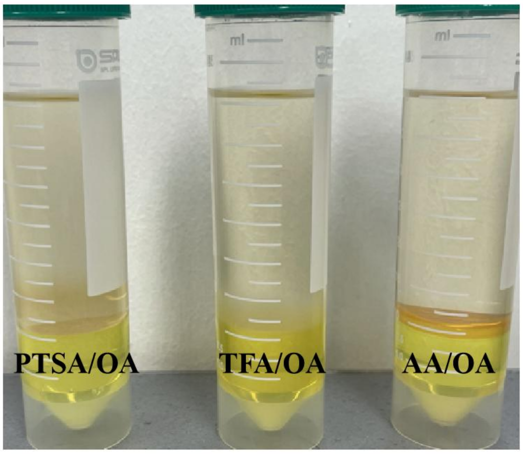

Supplementary Fig. 1 The digital images of three kinds of dual acid-mediated interfacial polymerization system.

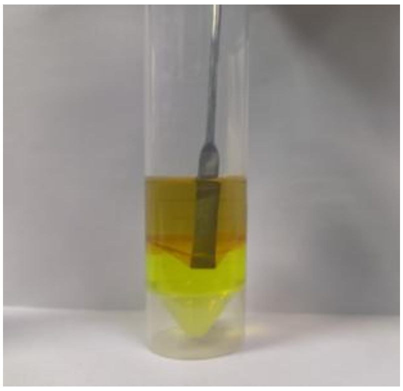

Supplementary Fig. 2 Photograph of the 3D SCOF membrane pressed with a spoon, showing the mechanical robustness of the membrane.

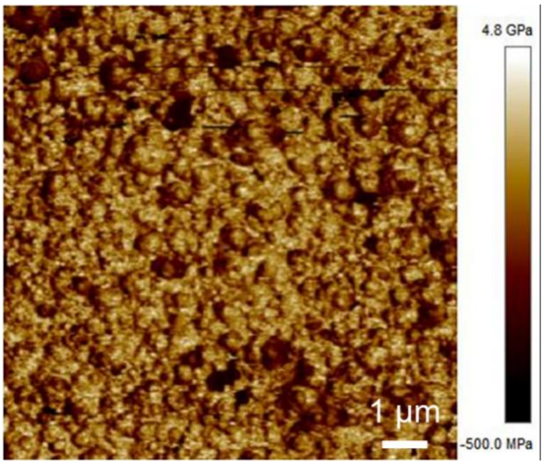

Supplementary Fig. 3 Young's modulus of the 3D SCOF membrane tested using the peak force quantitative nanomechanical property mapping method. The 3D SCOF membrane possessed a high Young's modulus of approximately 2.4 GPa on average.

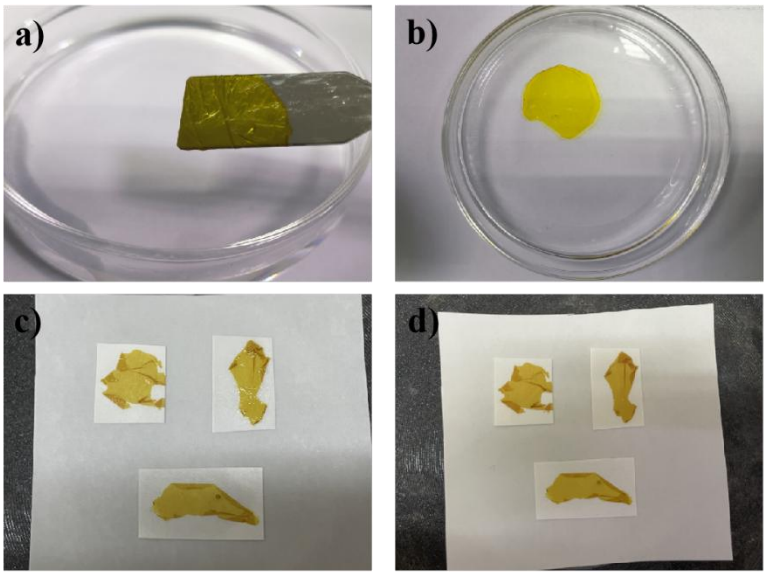

Supplementary Fig. 4 Photographs of the 3D SCOF membrane (a) on a spatula, (b) soaked in water and on a polyacrylonitrile substrate (c) before and (d) after drying. It can be observed that the membrane was still intact and there were no observable morphology changes.

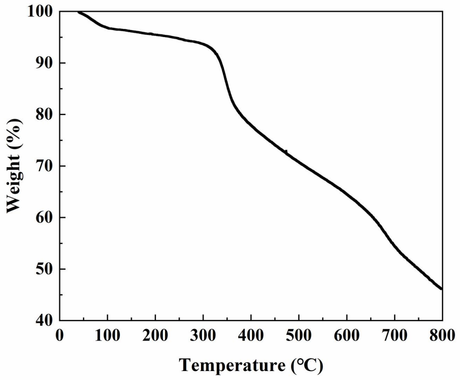

Supplementary Fig. 5 The TGA result of 3D SCOF membrane.

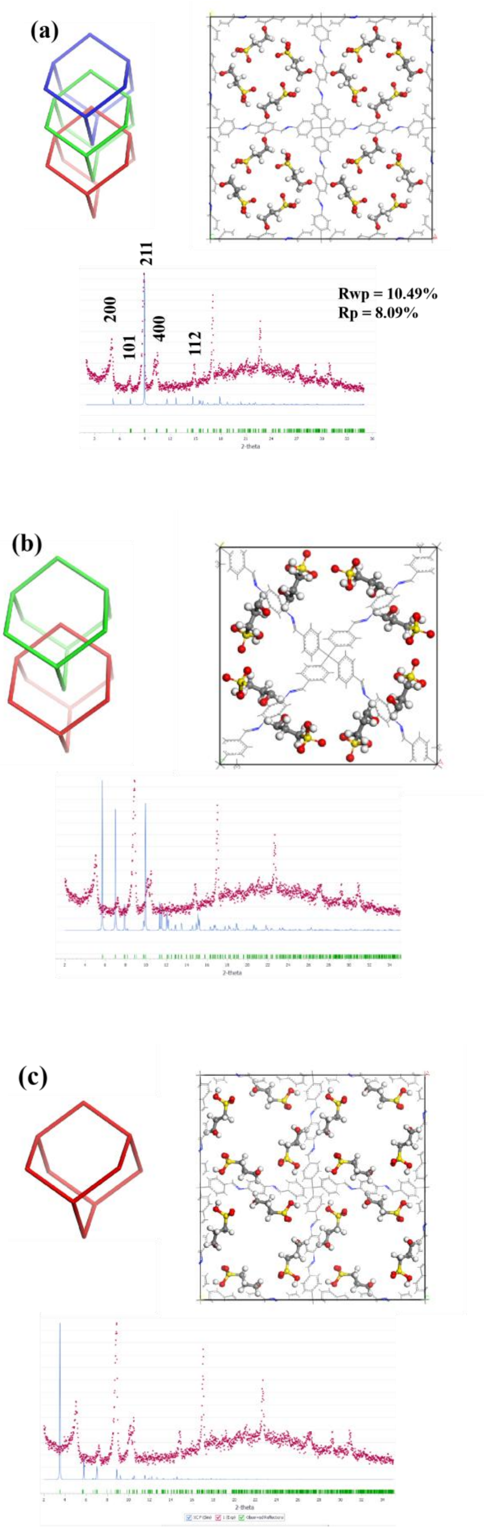

Supplementary Fig. 6 Indexed experimental (pink) and calculated (blue) PXRD patterns for (a) 3-fold, (b) 2-fold, and (c) 1-fold interpenetrated dia structure of 3D SCOF membrane.

Supplementary Table 1 Fractional atomic coordinates for the unit cell of 3D SCOF membrane (triple interpenetrating structure, Supplementary Fig. 6a).

Crystal system: Tetragonal; Space group: *I*41/*A* (No. 88)									
a=*b*=34.1017 Å; *c*=13.0803 Å; *α*=*β*=*γ*=90°									
Atom	*x*	*y*	*z*	occupancy	Atom	*x*	*y*	*z*	occupancy
C1	0.50000	0.50000	0.00000	1.00	H4	0.55793	0.93784	0.27522	1.00
C2	0.50147	0.96222	0.32267	1.00	H5	0.53927	0.81969	0.44623	1.00
C3	0.52867	0.04489	0.39516	1.00	H6	0.45889	0.80912	0.65107	1.00
C4	0.46956	0.91991	0.45333	1.00	H7	0.23201	0.59948	1.40198	1.00
C5	0.49822	0.89034	0.44478	1.00	H8	0.21886	0.61249	1.52932	1.00
C6	0.53014	0.89837	0.37939	1.00	H9	0.30305	0.61180	1.44092	1.00
C7	0.53196	0.93356	0.32098	1.00	H10	0.27382	0.65446	1.46573	1.00
C8	0.51512	0.82147	0.49972	1.00	H11	0.27509	0.64070	1.65316	1.00
C9	0.50718	0.78503	0.56462	1.00	H12	0.30050	0.59499	1.63122	1.00
C10	0.47688	0.78351	0.63789	1.00	H13	0.40291	0.64994	1.54774	1.00
C11	0.53056	0.75073	0.55350	1.00	O1	0.25060	0.56032	1.52030	1.00
C12	0.24279	0.59907	1.48288	1.00	O2	0.33927	0.69460	1.58027	1.00
C13	0.27999	0.62399	1.49183	1.00	O3	0.36129	0.65155	1.71216	1.00
C14	0.29573	0.62515	1.60161	1.00	O4	0.38005	0.63411	1.52601	1.00
H1	0.55196	0.02354	0.40401	1.00	N1	0.49387	0.85381	0.50408	1.00
H2	0.44523	0.91529	0.50513	1.00	S1	0.34292	0.65041	1.60429	1.00
H3	0.55411	0.87777	0.37381	1.00					

.

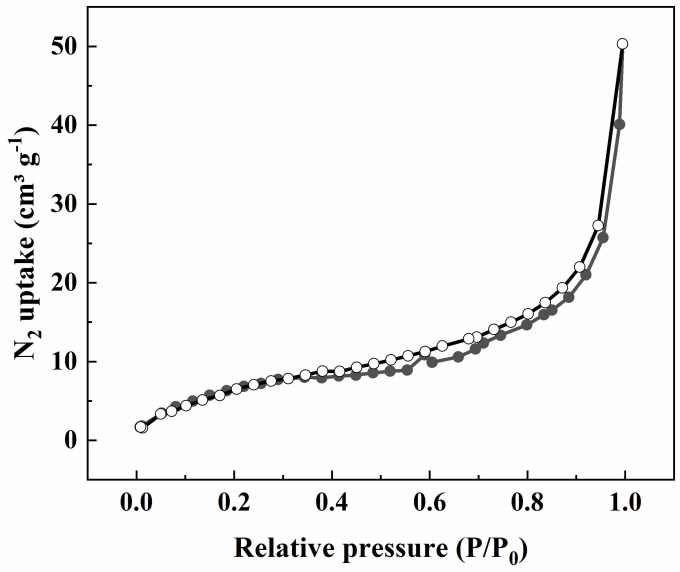

Supplementary Fig. 7 N_2 adsorption-desorption isotherms at 77K of 3D SCOF membrane.

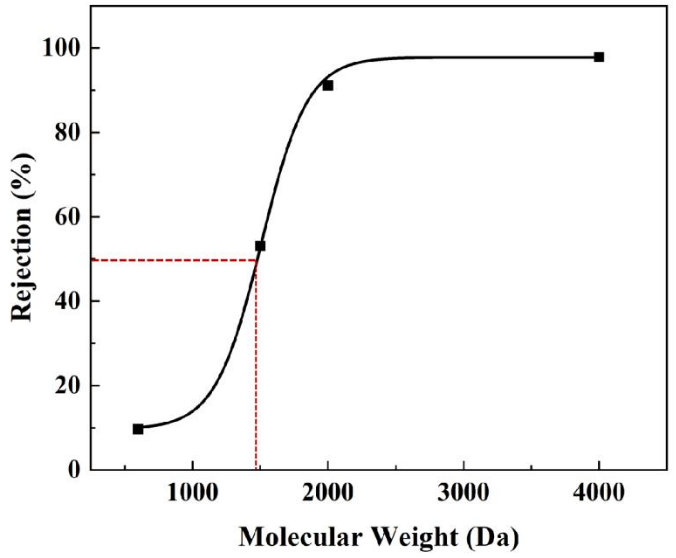

Supplementary Fig. 8 Molecular rejection of PEG by the 3D SCOF membrane as a function of molecular weight in the water system.

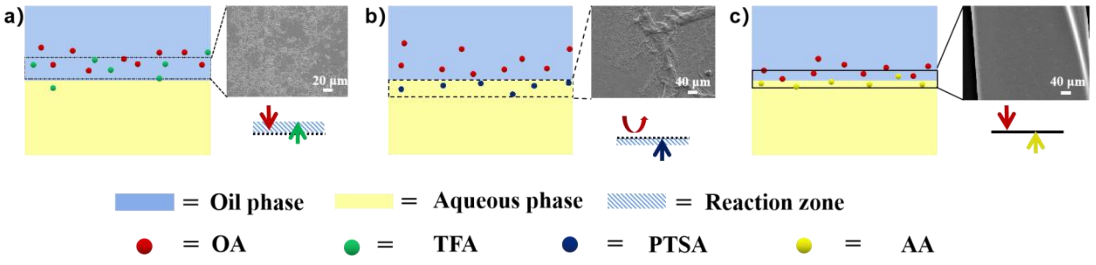

Supplementary Fig. 9 (a-c) Different interfacial polymerization processes under the following conditions: octanoic acid as the organic phase and trifluoroacetic acid, p-toluene sulfonic acid, or acetic acid solution as the aqueous phase respectively (TFA/OA, PTSA/OA and AA/OA systems).

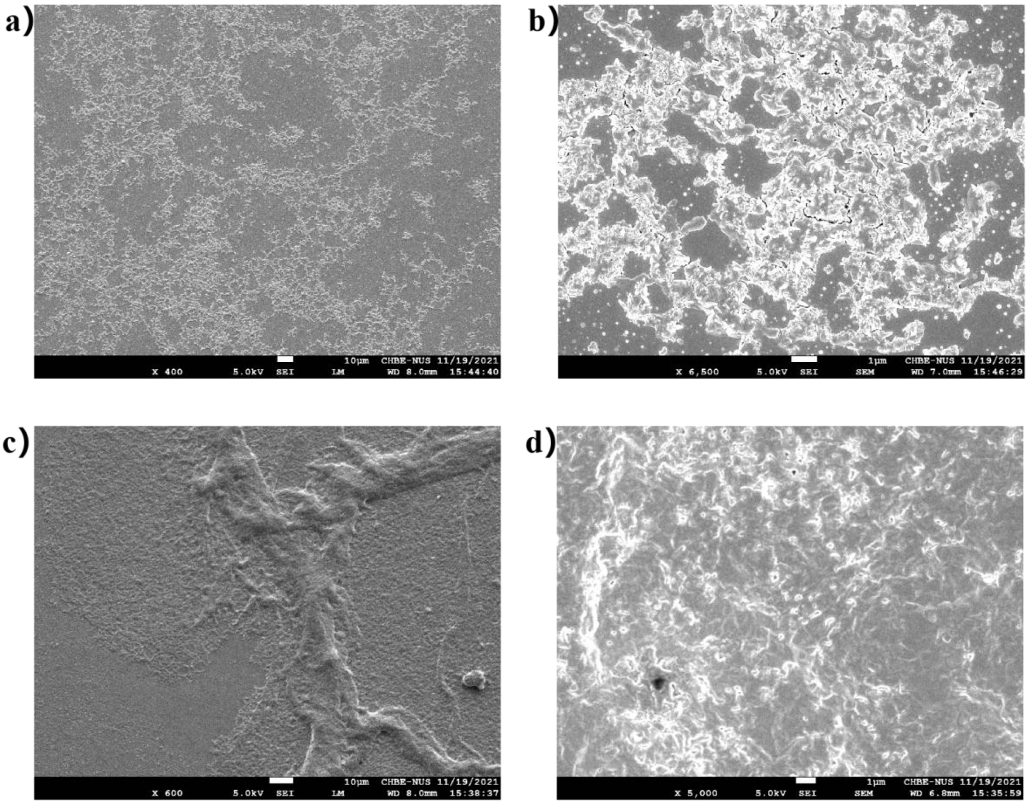

Supplementary Fig. 10 (a) and (b) The SEM images of TFA/OA system; (c) and (d), the SEM images of PTSA/OA system.

Fukui function calculation (indices for nucleophilic attack)

In this study, Fukui function was calculated for monomers reactivity discussed:

$$\text{Electrophilic reaction: } f_A^- = q_{N+1}^A - q_N^A \qquad (10)$$

$$\text{Nucleophilic reaction: } f_A^+ = q_N^A - q_{N-1}^A \qquad (11)$$

Where q_N^A is the charge of atom A in a molecule. In this work, the Materials Studio program was used for geometry optimizations, Mulliken charges calculation, and Hirshfeld charges calculation with the function of generalized gradient approximation (GGA) and Perdew-Burke-Ernzerhof (PBE). In general, the larger the value of the Fukui function at a reaction site, the higher the reactivity of that corresponding site[2]. It should be noted that some of the calculated results of chemical equivalent reaction sites are not equivalent. In this case, an average of results was taken and used for all equivalent reaction sites (the yellow mark are the calculated values of the reaction sites).

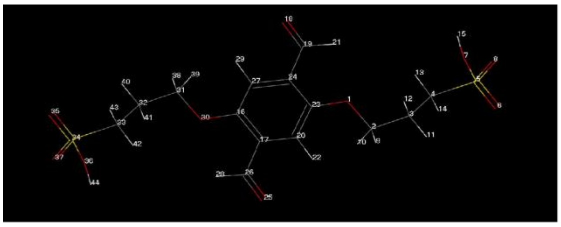

Fukui Indices for Nucleophilic Attack (Fukui(+))

atom	Mulliken	Hirshfeld	atom	Mulliken	Hirshfeld
O (1)	0.015	0.018	C (23)	0.036	0.034
C (2)	-0.031	0.006	C (24)	0.028	0.052
C (3)	-0.006	0.004	O (25)	0.109	0.112
C (4)	-0.01	-0.004	C (26)	0.077	0.09
S (5)	0.002	0.003	C (27)	0.05	0.051
O (6)	0.017	0.015	H (28)	0.069	0.042
O (7)	0.006	0.005	H (29)	0.048	0.026
O (8)	0.017	0.014	O (30)	0.015	0.018
H (9)	0.019	0.008	C (31)	-0.031	0.006
H (10)	0.02	0.008	C (32)	-0.006	0.004
H (11)	0.023	0.012	C (33)	-0.01	-0.004
H (12)	0.01	0.005	S (34)	0.002	0.003
H (13)	-0.012	-0.006	O (35)	0.017	0.015
H (14)	0.01	0.002	O (36)	0.006	0.005
H (15)	0.002	0.002	O (37)	0.017	0.014
C (16)	0.036	0.034	H (38)	0.019	0.008
C (17)	0.028	0.052	H (39)	0.02	0.008
O (18)	0.109	0.112	H (40)	0.023	0.012
C (19)	0.077	0.09	H (41)	0.01	0.005
C (20)	0.05	0.051	H (42)	-0.012	-0.006
H (21)	0.069	0.042	H (43)	0.01	0.002
H (22)	0.048	0.026	H (44)	0.002	0.002

Supplementary Fig. 11 The Fukui calculation result of monomer A.

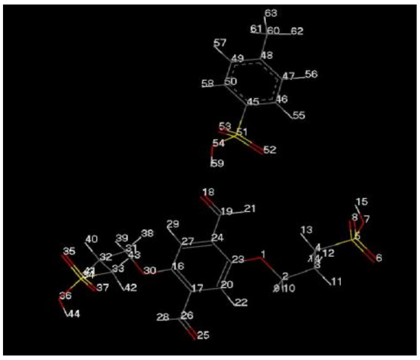

Fukui Indices for Nucleophilic Attack (Fukui(+))

atom	Mulliken	Hirshfeld						
O (1)	0.018	0.02	H (22)	0.046	0.025	H (43)	0.005	0
C (2)	-0.03	0.006	C (23)	0.041	0.036	H (44)	0.003	0.003
C (3)	-0.005	0.003	C (24)	0.011	0.041	C (45)	-0.004	-0.004
C (4)	-0.01	-0.004	O (25)	0.1	0.102	C (46)	-0.001	-0.001
S (5)	0.002	0.003	C (26)	0.063	0.077	C (47)	0.002	0.005
O (6)	0.016	0.014	C (27)	0.054	0.05	C (48)	0.003	0.008
O (7)	0.006	0.005	H (28)	0.063	0.038	C (49)	0.003	0.006
O (8)	0.016	0.014	H (29)	0.044	0.025	C (50)	-0.002	0.001
H (9)	0.023	0.01	O (30)	0.016	0.018	S (51)	-0.005	0
H (10)	0.017	0.007	C (31)	-0.028	0.006	O (52)	-0.006	-0.005
H (11)	0.022	0.011	C (32)	-0.005	0.004	O (53)	0.017	0.012
H (12)	0.009	0.004	C (33)	-0.01	-0.004	O (54)	0	-0.001
H (13)	-0.012	-0.005	S (34)	0.002	0.003	H (55)	-0.002	-0.001
H (14)	0.01	0.003	O (35)	0.015	0.013	H (56)	0.01	0.005
H (15)	0.002	0.002	O (36)	0.007	0.006	H (57)	0.013	0.006
C (16)	0.03	0.03	O (37)	0.016	0.013	H (58)	0.003	0.001
C (17)	0.035	0.055	H (38)	0.013	0.006	H (59)	-0.007	0.002
O (18)	0.098	0.096	H (39)	0.021	0.008	C (60)	-0.003	0.003
C (19)	0.101	0.102	H (40)	0.02	0.011	H (61)	0.006	0.004
C (20)	0.044	0.048	H (41)	0.013	0.006	H (62)	0.007	0.004
H (21)	0.062	0.041	H (42)	-0.009	-0.005	H (63)	0.01	0.006

Supplementary Fig. 12 The Fukui calculation result of monomer A with PTSA.

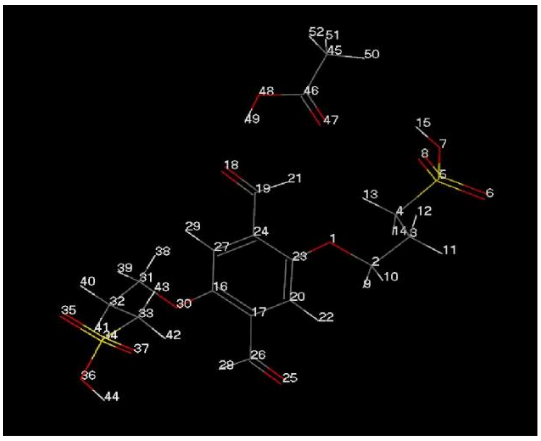

Fukui Indices for Nucleophilic Attack (Fukui(+))					
atom	Mulliken	Hirshfeld			
O (1)	0.016	0.019	C (27)	0.053	0.051
C (2)	-0.03	0.006	H (28)	0.066	0.04
C (3)	-0.005	0.004	H (29)	0.047	0.026
C (4)	-0.009	-0.003	O (30)	0.015	0.018
S (5)	0.002	0.003	C (31)	-0.029	0.006
O (6)	0.017	0.015	C (32)	-0.006	0.004
O (7)	0.003	0.003	C (33)	-0.01	-0.004
O (8)	0.017	0.014	S (34)	0.002	0.003
H (9)	0.023	0.01	O (35)	0.016	0.014
H (10)	0.017	0.007	O (36)	0.007	0.006
H (11)	0.023	0.012	O (37)	0.016	0.013
H (12)	0.008	0.004	H (38)	0.014	0.006
H (13)	-0.013	-0.006	H (39)	0.023	0.009
H (14)	0.012	0.004	H (40)	0.022	0.011
H (15)	0.001	0.001	H (41)	0.013	0.006
C (16)	0.033	0.032	H (42)	-0.011	-0.005
C (17)	0.032	0.053	H (43)	0.005	0
O (18)	0.098	0.096	H (44)	0.003	0.002
C (19)	0.084	0.092	C (45)	-0.003	0.008
C (20)	0.047	0.05	C (46)	0.001	0.006
H (21)	0.058	0.034	O (47)	-0.012	-0.005
H (22)	0.047	0.026	O (48)	0.015	0.011
C (23)	0.037	0.035	H (49)	-0.002	0.003
C (24)	0.02	0.046	H (50)	0.013	0.008
O (25)	0.104	0.106	H (51)	0.017	0.01
C (26)	0.07	0.083	H (52)	0.015	0.009

Supplementary Fig. 13 The Fukui calculation result of monomer A with AA.

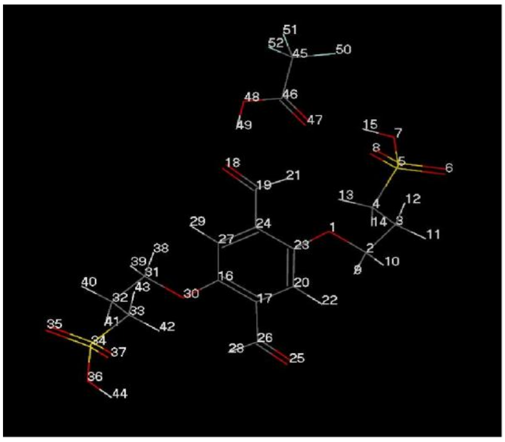

Fukui Indices for Nucleophilic Attack (Fukui(+))					
atom	Mulliken	Hirshfeld	atom	Mulliken	Hirshfeld
O (1)	0.019	0.021	C (27)	0.056	0.051
C (2)	-0.031	0.006	H (28)	0.063	0.037
C (3)	-0.007	0.004	H (29)	0.046	0.025
C (4)	-0.007	-0.003	O (30)	0.016	0.018
S (5)	0.002	0.003	C (31)	-0.029	0.005
O (6)	0.021	0.018	C (32)	-0.006	0.004
O (7)	0.005	0.004	C (33)	-0.009	-0.004
O (8)	0.016	0.014	S (34)	0.002	0.003
H (9)	0.02	0.008	O (35)	0.016	0.014
H (10)	0.024	0.01	O (36)	0.005	0.005
H (11)	0.026	0.013	O (37)	0.016	0.014
H (12)	0.01	0.005	H (38)	0.017	0.007
H (13)	-0.021	-0.008	H (39)	0.019	0.008
H (14)	0.016	0.005	H (40)	0.021	0.011
H (15)	0	0	H (41)	0.011	0.005
C (16)	0.029	0.03	H (42)	-0.012	-0.005
C (17)	0.038	0.057	H (43)	0.009	0.002
O (18)	0.096	0.097	H (44)	0.002	0.002
C (19)	0.108	0.103	C (45)	0.009	0.005
C (20)	0.044	0.048	C (46)	0.003	0.006
H (21)	0.051	0.034	O (47)	-0.01	-0.003
H (22)	0.047	0.026	O (48)	0.011	0.009
C (23)	0.041	0.037	H (49)	0.002	0.007
C (24)	0.007	0.039	F (50)	0.009	0.009
O (25)	0.099	0.1	F (51)	0.009	0.009
C (26)	0.06	0.075	F (52)	0.01	0.01

Supplementary Fig. 14 The Fukui calculation result of monomer A with TFA.

Interaction Energy Calculation

The interaction energies between different acids and two monomers were carried out with Material Studio software through the first-principles density functional theory (DFT) approach. All calculations were carried out using DMol3 module with the basis set of dynamic nuclear polarization (DNP) and the functional of GGA/PBE. The binding energy ($\Delta E_{binding}$) between methyl phosphate and metal ion was calculated as follows:

$$\Delta E_{binding} = E_{(mix)} - E_{(monomer)} - E_{(Acid)} \qquad (12)$$

Supplementary Table 2. The interaction energy calculation results of monomers with different acid.

	Single-Energy (Ha)	Combined-Energy (Ha)	delta (Ha)	Delta (eV)	delta (kJ/mol)
Monomer A	-2091.68				
TFA	-526.53	-2618.23	-0.024	-0.65	-62.42
AA	-228.92	-2320.61	-0.012	-0.33	-31.66
PTSA	-894.88	-2986.56	-0.0086	-0.24	-22.69
Monomer B	-1184.93				
OA	-464.50	-1649.44	-0.012	-0.33	-32.18
NA	-503.83	-1688.71	0.051	1.38	133.35
HA	-386.01	-1570.92	0.028	0.75	72.81

Diffusion calculation (video)

A 3.7 nm × 3.7 nm × 7.8 nm simulation box was composed of 10 acids molecules (Acetic acid, Trifluoroacetate, p-Toluene sulfonic acid), 200 octanoic acid molecules, and 1718 water molecules. The interactions of all atoms were described by the 12-6 Lennard-Jones (LJ) and Columbic potentials:

$$\text{Unonbonded} = \sum 4\varepsilon_{ij}\left[\left(\frac{\sigma_{ij}}{r_{ij}}\right)^{12} - \left(\frac{\sigma_{ij}}{r_{ij}}\right)^{6}\right] + \sum \frac{q_i q_j}{4\pi\varepsilon_0 r_{ij}} \qquad (13)$$

where ε_{ij} and σ_{ij} are the strength and the diameter of LJ potential, r_{ij} is the distance between atom i and j, q_i is the charge of atom i and ε_0 is the vacuum permittivity. With the help of LigParGen web server[3], the forcefield parameters of the molecules were adopted from the OPLS/AA force field[4], and atomic charges were calculated by 1.14*CM1A model. In this work, we employed the TIP4P water model. All MD simulations were performed using GROMACS 5.1.4[5]. The periodic boundary conditions were applied along the three directions. We used 1.2 nm of cut off distance for LJ interactions and the particle-mesh Ewald method for electrostatic interactions with a grid spacing of 0.12 nm. The system was first energy minimized using the steepest descent method. After that the velocities of the system were assigned at 303.15 K using the V-rescale thermostat. The Parrinello-Rahman barostat was used to semi-isotopically regulate pressure on the *z*-direction. Finally, equilibrium MD simulation was run for 20 ns. The initial 5 ns was used to ensure the system reached equilibrium and the data were averaged over the last 15 ns. For each simulation, we used a time step of 2 fs and the trajectory was saved every 4 ps.

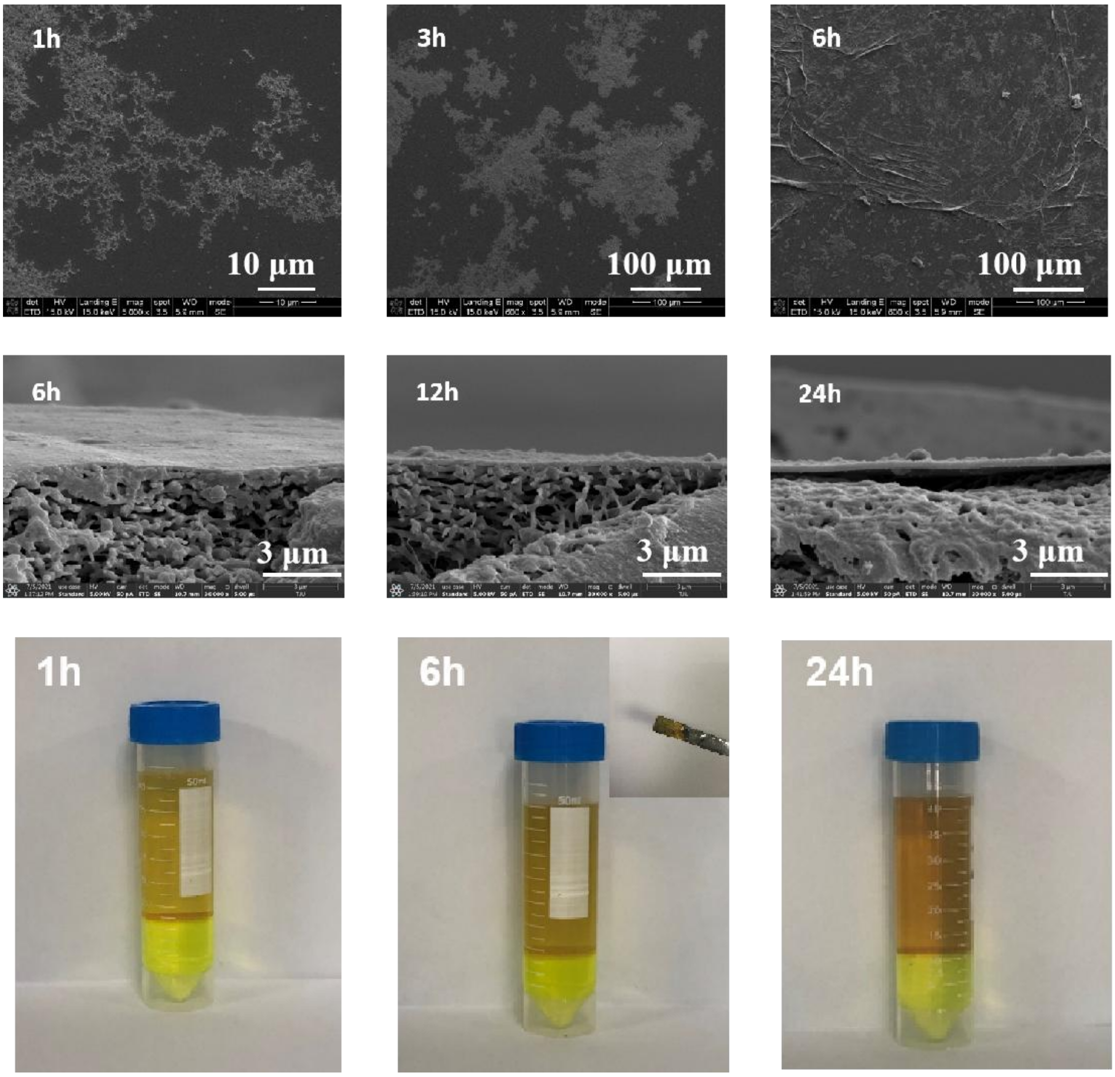

Supplementary Fig. 15 Images of the formation process of 3D SCOF membrane (3D SCOFM-AA/OA).

As the reaction time increases, particles are first formed between monomers, and the particles are connected to form clusters and then grow into a continuous membrane. The upper and lower two-phase solutions remain transparent and clear with the increase of reaction time.

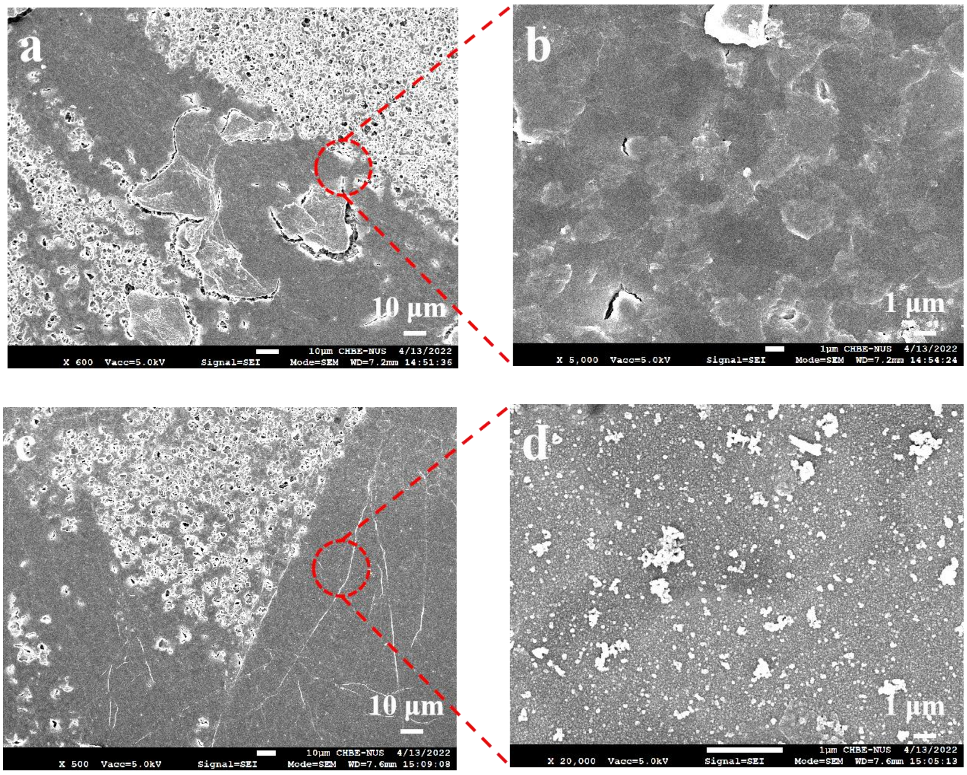

Supplementary Fig. 16 The SEM images of membranes prepared by the dual acid system. (a-b) AA/ HA; (c-d) AA / DA.

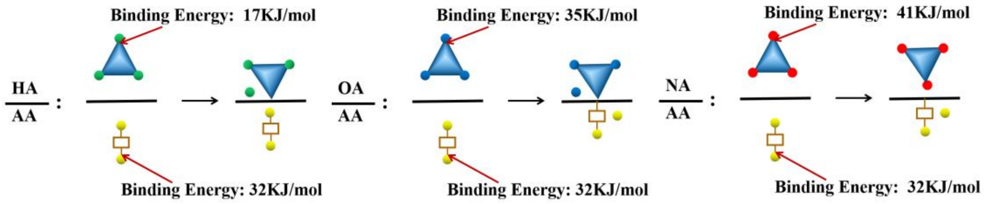

Supplementary Fig. 17 Interaction energies of the monomer B with three kinds of acids and monomer A with acetic acid.

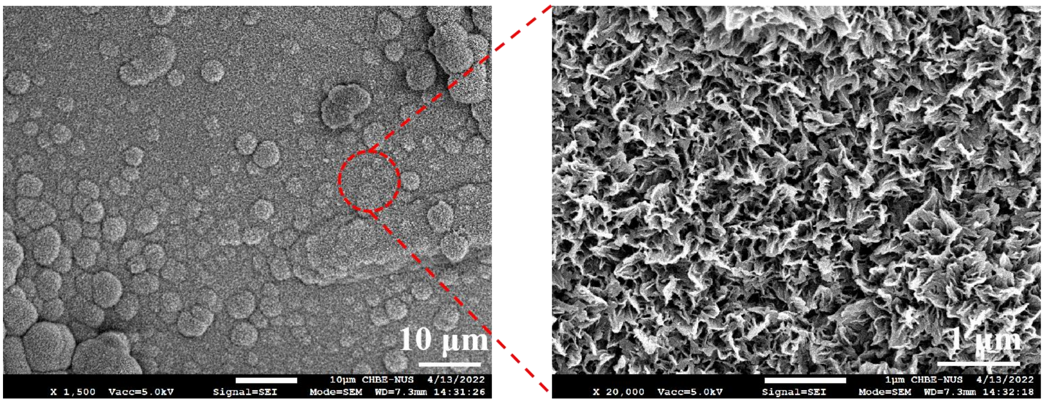

Supplementary Fig. 18 The SEM images of membrane prepared by the single acid system (mesitylene / acetic acid aqueous solution).

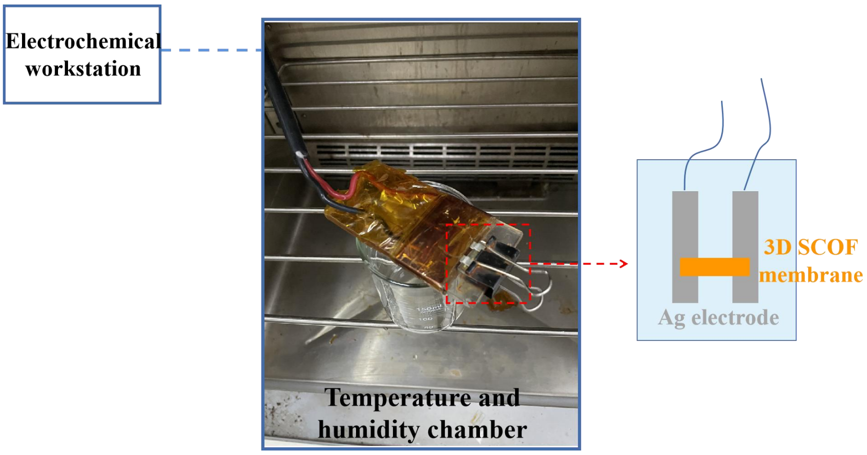

Supplementary Fig. 19 Schematic illustration of the experimental setup device for proton conductivity measurement.

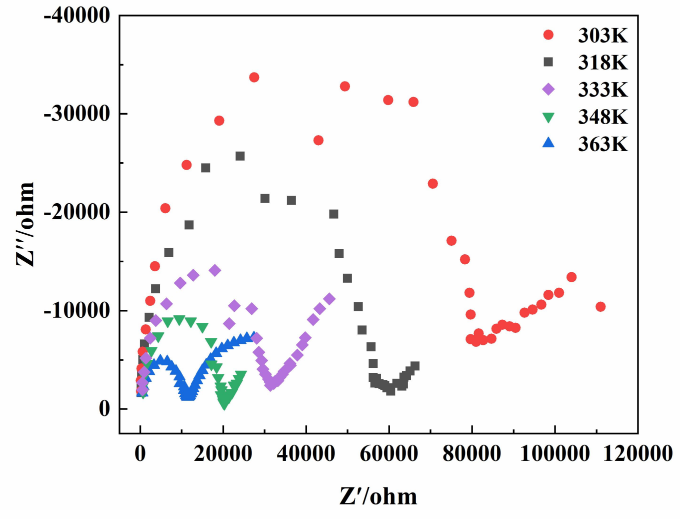

Supplementary Fig. 20 Nyquist plots of 3D SCOF membrane measured at different temperatures under 100% RH.

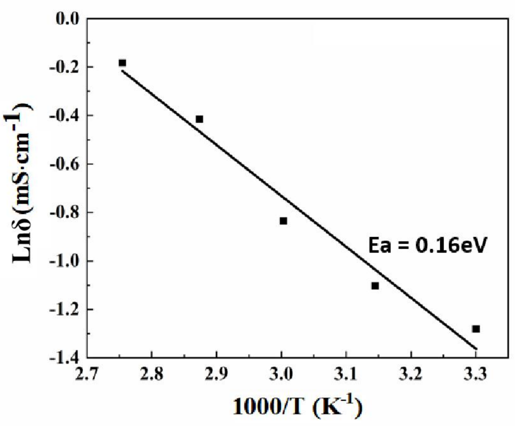

Supplementary Fig. 21 Arrhenius plot of the 3D SCOF membrane.

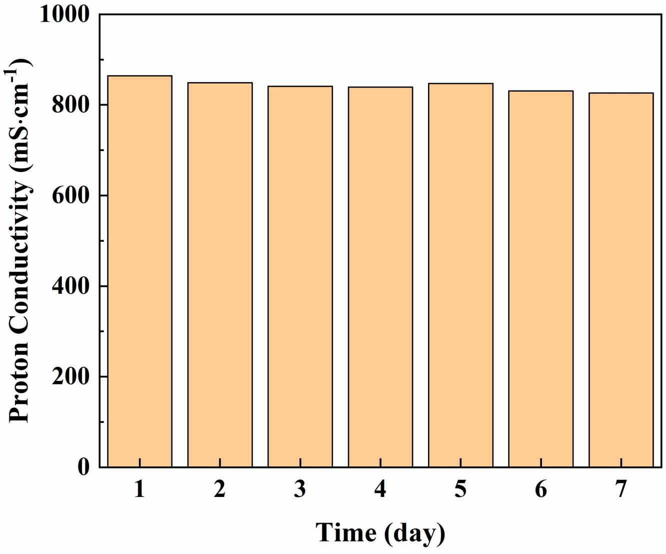

Supplementary Fig. 22 The long-term stability of 3D SCOF membrane in proton conduction at 60°C, 100% RH.

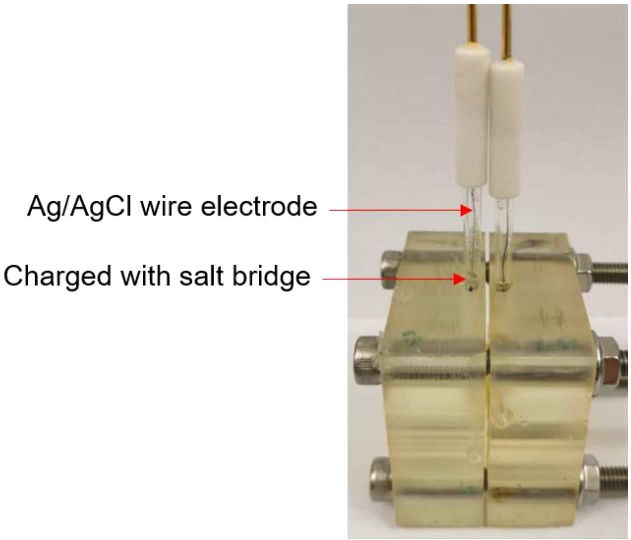

Supplementary Fig. 23 Photograph of the experimental setup device for current-voltage (*I*−*V*) measurement. The device is connected to the external detection equipment for collecting output current and voltage.

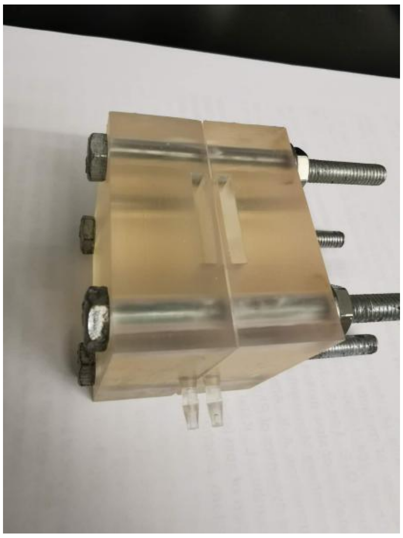

Supplementary Fig. 24 Photograph of the experimental setup device for osmotic energy conversion performance test. The device is connected to the external detection equipment for collecting output current and voltage.

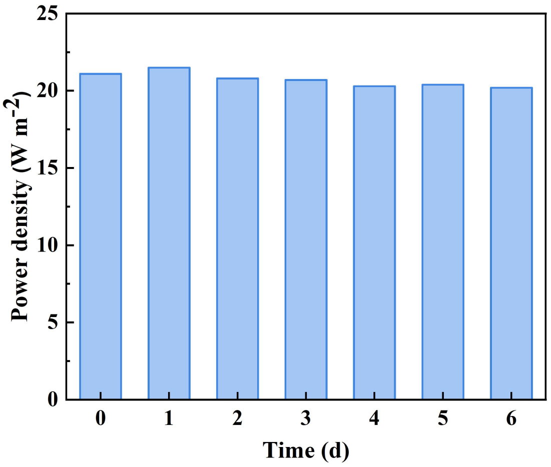

Supplementary Fig. 25 The long-term stability of 3D SCOF membrane in osmotic energy conversion under the standard artificial river water and sea water system.

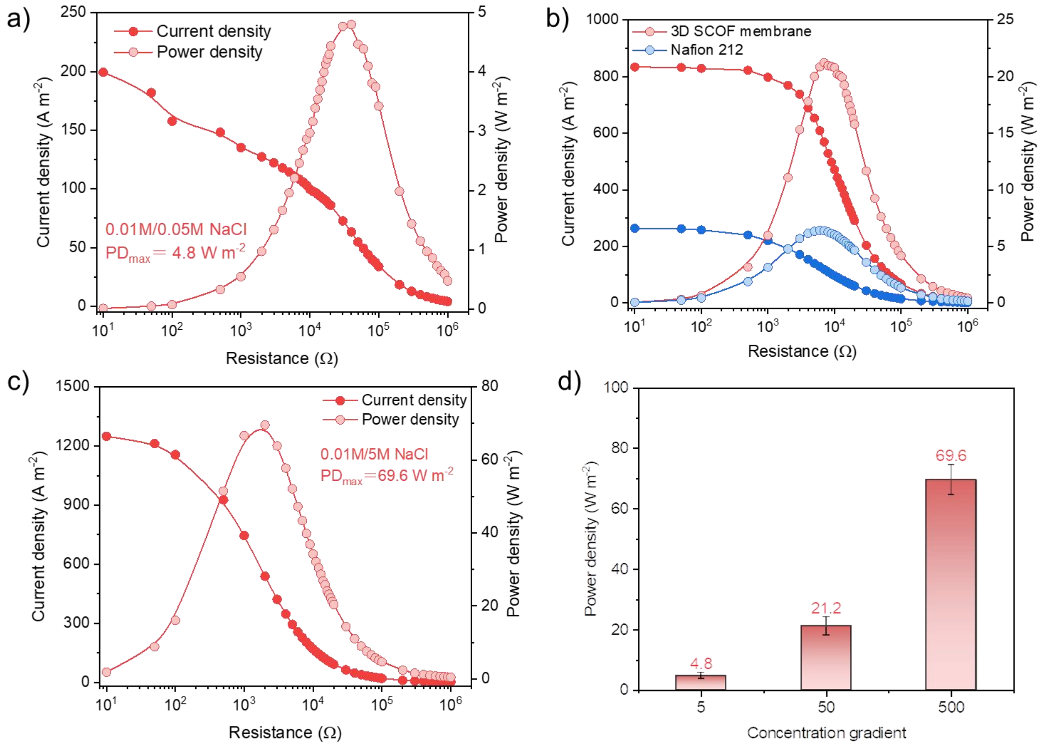

Supplementary Fig. 26 (a) The current density and power density of 3D SCOF membrane as functions of external resistances under a 5-fold concentration gradient; (b) Current density and power density of 3D SCOF and Nafion membranes as functions of external resistances under a 50-fold concentration gradient; (c) Current density and power density of 3D SCOF membrane as functions of external resistances under a 500-fold concentration gradient; (d) Output power density of the 3D SCOF membranes under different concentration gradient. The error bars in this figure represent standard deviations for three measurements.

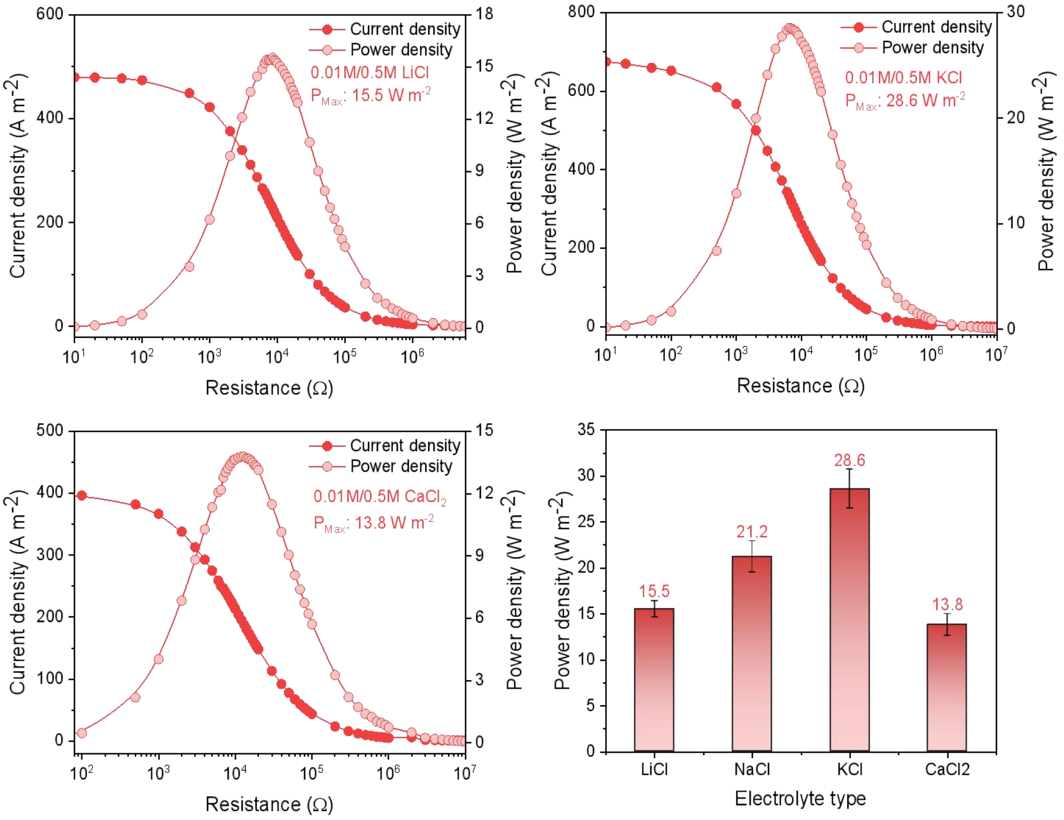

Supplementary Fig. 27 The output power density of the 3D SCOF membranes under other typical types of chloride salt solutions. The error bars in this figure represent standard deviations for three measurements.

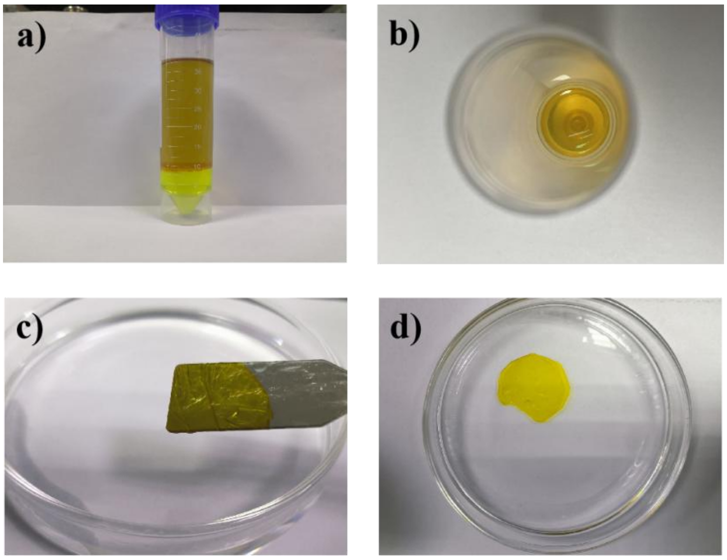

Supplementary Fig. 28 Photographs of (a) the interfacial reaction system, (b) the 3D SCOF membrane on the water solution surface, (c) on a spatula, and (d) in ethanol solvent.

Supplementary Table 3. The proton conducting materials in Fig. 3b.

Proton conducting materials	Category	Performance (mS cm^{-1})	Ref.
COF-F6-H-P	COF powder	14.1 (353K, 0% RH)	6
NUS-10(R)-P	COF powder	39.6(298K, 100% RH)	7
H3PO4@NKCOF-1	COF powder	113 (333K, 100% RH)	8
Etidronic acid@COF-300	COF membrane	650 (363K, 100% RH)	9
IPC-COF-M	COF membrane	380 (353K, 100% RH)	10
IM-UiO-66-AS	MOF powder	154 (363K, 100% RH)	11
BUT-8-(Cr)A	MOF powder	127 (353K, 100% RH)	12
PSM-1	MOF powder	164 (353K, 100% RH)	13
SPX-BP-0.95	PIM membrane	180 (353K, 100% RH)	14
SPES/CC3	PEM	315 (353K, 100% RH)	15
SPS/MOF	PEM	212 (353K, 100% RH)	16
SPEEK/MOF	PEM	306 (353K, 100% RH)	17
GO/SCA-30%	PEM	327 (363K, 100% RH)	18
Nafion/Z-COF	PEM	220 (353K, 100% RH)	19
Nafion-117	PEM	179 (353K, 100% RH)	20
Sulfonated ABA triblock PES copolymer	PEM	210 (353K, 100% RH)	21
PAEK containing sulfonated side chain	PEM	271 (353K, 100% RH)	22
Cross-linked sulfonated PAEK	PEM	221 (353K, 100% RH)	23
3D SCOFM	COF membrane	843 (353K, 100% RH)	This work!

Supplementary Table 4. The osmotic energy conversion performance of previously reported membranes.

Materials	Channel size (nm)	Power density (W m^{-2}, NaCl)	Energy conversion efficiency (%)	Ref.
SPX membrane	0.5-0.7	1.23 (50-fold)	37.1	24
$Ti_3C_2T_X$Mxene	0.64	21.00 (1000-fold)	40.6	25
GO/SNF/GO	0.75	5.07 (50-fold)	14.6	26
SPEEK membrane	2.7	5.80 (50-fold)	16.7	27
CMWs	3.5	2.78 (50-fold)	36.3	28
Hydrogel membrane	7	5.38 (50-fold)	14.5	29
Janus membrane	M-1:10/M-2:17	2.10 (50-fold)	17.5	30
ABN30 membrane	-	7.70 (50-fold)	5.9	31
MOF/SPEEK	-	7.00 (50-fold)	40.0	32
Bipolar membrane	-	6.20 (50-fold)	-	33
Phosphate hydrogel membrane	-	5.38 (50-fold)	-	34
Nanochannel array membrane	8.4-18.2	22.4 (500-fold)	-	35
Polyelectrolyte hydrogel	-	7.87 (50-fold)	-	36
Vertical-aligned BN nanopores	30	106 (1000-fold)	12.0	37
Vertical-aligned GO	0.86	10.60 (50-fold)	18.7	38
Vertical-aligned Mxene	0.68-1.07	4.6 (50-fold)	44.2	39
COF−SO3H− 24/ COF−QA−24	2-2.5	15.2 (1μm membrane, 50-fold)	-	40
BDA-TAM COF	1.4	5.31 (KCL, 50-fold)	28.3	41
COF-LZU1	1.8	4.26 (50-fold)	-	42
TFP-TPA COF@ANM	1.1	27.8 (500-fold)	45.3	43
TpEB@TpPa-SO3Na	1-2	19.2 (50-fold)	26.9	44
PyPa-SO3H/SANF	2.3	9.6 (50-fold)	-	45
3D SCOF membrane	0.97	21.2 (50-fold)	45.3	This Work!

Reference

1. Zachariah, A. P. *et al.* Hydrophilic Conjuated Polymer Prepared by Aqueous Horner-Wadsworth-Emmons Coupling. *Macromolecules* **49**, 2526–2532 (2016).
2. Perdew, J. P., Burke, K. & Ernzerhof, M. Generalized Gradient Approximation Made Simple. *Physical Review Letters* **77**, 3865–3868 (1996).
3. Dodda, L. S., Vilseck, J. Z., Tirado-Rives, J. & Jorgensen, W. L. 1.14*CM1A-LBCC: Localized Bond-Charge Corrected CM1A Charges for Condensed-Phase Simulations. *Journal of Physical Chemistry B* **121**, 3864–3870 (2017).
4. Jorgensen, W. L., Maxwell, D. S. & Tirado-Rives, J. Development and Testing of the OPLS All-Atom Force Field on Conformational Energetics and Properties of Organic Liquids. *Journal of the American Chemical Society* **118**, 11225–11236 (1996).
5. Van Der Spoel, D. *et al.* GROMACS: Fast, flexible, and free. *Journal of Computational Chemistry* **26**, 1701–1718 (2005).
6. Liu, X. *et al.* Recent advances in covalent organic frameworks (COFs) as a smart sensing material. *Chemical Society Reviews* **48**, 5266–5302 (2019).
7. Peng, Y. *et al.* Mechanoassisted Synthesis of Sulfonated Covalent Organic Frameworks with High Intrinsic Proton Conductivity. *ACS Applied Materials Interfaces* **8**, 18505–18512 (2016).
8. Yang, Y. *et al.* Combined Intrinsic and Extrinsic Proton Conduction in Robust Covalent Organic Frameworks for Hydrogen Fuel Cell Applications. *Angewandte Chemie - International Edition* **59**, 3678–3684 (2020).
9. Fan, C. *et al.* Three-dimensional covalent organic framework membrane for efficient proton conduction. *Journal of Materials Chemistry* A **9**, 17720–17723 (2021).
10. Cao, L. *et al.* Weakly Humidity-Dependent Proton-Conducting COF Membranes. *Advanced Materials* **32**, 2005565 (2020).

11. Wang, S. *et al.* Amino acid-functionalized metal organic framework with excellent proton conductivity for proton exchange membranes. *International Journal of Hydrogen Energy* **46**, 1163–1173 (2021).
12. Mukhopadhyay, S. *et al.* Designing UiO-66-Based Superprotonic Conductor with the Highest Metal–Organic Framework Based Proton Conductivity. *ACS Applied Materials Interfaces* **11**, 13423–13432 (2019).
13. Li, X.-M. *et al.* Synergistic Conductivity Effect in a Proton Sources-Coupled Metal–Organic Framework. *ACS Energy Letters* **2**, 2313–2318 (2017).
14. Zuo, P. *et al.* Sulfonated Microporous Polymer Membranes with Fast and Selective Ion Transport for Electrochemical Energy Conversion and Storage. *Angewandte Chemie - International Edition* **59**, 9564–9573 (2020).
15. Xu, X. *et al.* Enhancing proton conductivity of proton exchange membrane with SPES nanofibers containing porous organic cage. *Polymers for Advanced Technologies* **31**, 1571–1580 (2020).
16. Yin, Z. *et al.* Improved proton conduction of sulfonated poly (ether ether ketone) membrane by sulfonated covalent organic framework nanosheets. *International Journal of Hydrogen Energy* **46**, 26550–26559 (2021).
17. Li, Z. *et al.* Enhanced proton conductivity of proton exchange membranes by incorporating sulfonated metal-organic frameworks. *Journal of Power Sources* **262**, 372–379 (2014).
18. Mao, X. *et al.* Supramolecular Calix[n]arenes-Intercalated Graphene Oxide Membranes for Efficient Proton Conduction. *ACS Applied Materials Interfaces* **11**, 42250–42260 (2019).
19. Li, Y. *et al.* Fabrication of Nafion/zwitterion-functionalized covalent organic framework composite membranes with improved proton conductivity. *Journal of Membrane Science* **568**, 1–9 (2018).
20. He, G. *et al.* Nanostructured Ion-Exchange Membranes for Fuel Cells: Recent Advances and Perspectives. *Advanced Materials* **27**, 5280–5295 (2015).
21. Shin, D. W., Guiver, M. D. & Lee, Y. M. Hydrocarbon-Based Polymer Electrolyte Membranes: Importance of Morphology on Ion Transport and

Membrane Stability. *Chemical Reviews* **117**, 4759–4805 (2017).

22. Cao, L. *et al.* Channel-facilitated molecule and ion transport across polymer composite membranes. *Chemical Society Reviews* **46**, 6725–6745 (2017).
23. Shi, B. *et al.* Control of Edge/in-Plane Interactions toward Robust, Highly Proton Conductive Graphene Oxide Membranes. *ACS Nano* **13**, 10366–10375 (2019).
24. Zhu, Q. *et al.* A sulfonated ultramicroporous membrane with selective ion transport enables osmotic energy extraction from multiform salt solutions with exceptional efficiency. *Energy Environment Science* **15**, 4148–4156 (2022).
25. Hong, S. *et al.* Two-Dimensional $Ti_3C_2T_x$ Mxene Membranes as Nanofluidic Osmotic Power Generators. *ACS Nano* **13**, 8917–8925 (2019).
26. Xin, W. *et al.* Biomimetic Nacre-Like Silk-Crosslinked Membranes for Osmotic Energy Harvesting. *ACS Nano* **14**, 9701–9710 (2020).
27. Zhao, Y. *et al.* Robust sulfonated poly (ether ether ketone) nanochannels for high-performance osmotic energy conversion. *National Science Review* **7**, 1349–1359 (2020).
28. Xie, L. *et al.* Sequential Superassembly of Nanofiber Arrays to Carbonaceous Ordered Mesoporous Nanowires and Their Heterostructure Membranes for Osmotic Energy Conversion. *Journal of the American Chemical Society* **143**, 6922–6932 (2021).
29. Chen, W. *et al.* Improved Ion Transport and High Energy Conversion through Hydrogel Membrane with 3D Interconnected Nanopores. *Nano Letters* **20**, 5705–5713 (2020).
30. Zhang, Z. *et al.* Ultrathin and Ion-Selective Janus Membranes for High-Performance Osmotic Energy Conversion. J *Journal of the American Chemical Society* **139**, 8905–8914 (2017).
31. Liu, J. *et al.* Self-standing and flexible covalent organic framework (COF) membranes for molecular separation. *Science Advances* **6**, eabb1110 (2020).
32. Zhao, X. *et al.* Metal organic framework enhanced SPEEK/SPSF heterogeneous membrane for ion transport and energy conversion. *Nano*

Energy **81**, 105657 (2021).

33. Sun, Y. *et al.* Tailoring A Poly (ether sulfone) Bipolar Membrane: Osmotic-Energy Generator with High Power Density. *Angewandte Chemie - International Edition* **59**, 17423–17428 (2020).
34. Chen, W. *et al.* Improved Ion Transport and High Energy Conversion through Hydrogel Membrane with 3D Interconnected Nanopores. *Nano Letters* **20**, 5705–5713 (2020).
35. Li, C. *et al.* Large-scale, robust mushroom-shaped nanochannel array membrane for ultrahigh osmotic energy conversion. *Science Advances* **7**, eabg2183 (2021).
36. Bian, G. *et al.* Anti-Swelling Gradient Polyelectrolyte Hydrogel Membranes as High-Performance Osmotic Energy Generators. *Angewandte Chemie - International Edition* **60**, 20294–20300 (2021).
37. Pendse, A. *et al.* Highly Efficient Osmotic Energy Harvesting in Charged Boron-Nitride-Nanopore Membranes. *Advanced Functional Materials* **31**, 2009586 (2021).
38. Zhang, Z. *et al.* Vertically Transported Graphene Oxide for High-Performance Osmotic Energy Conversion. *Science Advances* **7**, 2000286 (2020).
39. Ding, L. *et al.* Oppositely Charged $Ti_3C_2T_x$ Mxene Membranes with 2D Nanofluidic Channels for Osmotic Energy Harvesting. *Angewandte Chemie - International Edition* **59**, 8720–8726 (2020).
40. Cao, L. *et al.* Giant Osmotic Energy Conversion through Vertical-Aligned Ion-Permselective Nanochannels in Covalent Organic Framework Membranes. *Journal of the American Chemical Society* **144**, 12400–12409 (2022).
41. Wang, C. *et al.* Ultrathin Self-Standing Covalent Organic Frameworks toward Highly-Efficient Nanofluidic Osmotic Energy Generator. *Advanced Functional Materials* **32**, 2204068 (2022).
42. Li, R., Zhai, J., Jiang, J., Wang, Q. & Wang, S. Improved Interfacial Ion Transport through Nanofluidic Hybrid Membranes Based on Covalent Organic Frameworks for Osmotic Energy Generation. *ACS Applied Energy Materials* **5**,

7176–7184 (2022).

43. Gao, M. *et al.* A bioinspired ionic diode membrane based on sub-2 nm covalent organic framework channels for ultrahigh osmotic energy generation. *Nano Energy* **105**, 108007 (2023).

44. Cao, L. *et al.* An Ionic Diode Covalent Organic Framework Membrane for Efficient Osmotic Energy Conversion. *ACS Nano* **16**, 18910–18920 (2022).

45. Man, Z. *et al.* Serosa-Mimetic Nanoarchitecture Membranes for Highly Efficient Osmotic Energy Generation. *Journal of the American Chemical Society* **143**, 16206–16216 (2021).
