# Supplementary material for: 3D covalent organic framework membrane with fast and selective ion transport
Source: Nat Commun. 2023 Sep 22;14:5926. doi: 10.1038/s41467-023-41555-5 (PMC10517170; doi:10.1038/s41467-023-41555-5)
Supplement: Supplementary file 3 — Description of Additional Supplementary Files [file 41467_2023_41555_MOESM3_ESM.pdf]

Description of Additional Supplementary Files

File Name: Supplementary Movie 1

Description: Simulation on diffusion behavior of trifluoroacetic acid (TFA) in the biphasic system. A 3.7 nm × 3.7 nm × 7.8 nm simulation box was composed of 10 acids molecules, 200 octanoic acid molecules, and 1718 water molecules. Equilibrium MD simulation was run for 20 ns. The initial 5 ns was used to ensure the system reached equilibrium and the data were averaged over the last 15 ns. For each simulation, we used a time step of 2 fs and the trajectory was saved every 4 ps.

File Name: Supplementary Movie 2

Description: Simulation on diffusion behavior of acetic acid (AA) in the biphasic system. A 3.7 nm × 3.7 nm × 7.8 nm simulation box was composed of 10 acids molecules, 200 octanoic acid molecules, and 1718 water molecules. Equilibrium MD simulation was run for 20 ns. The initial 5 ns was used to ensure the system reached equilibrium and the data were averaged over the last 15 ns. For each simulation, we used a time step of 2 fs and the trajectory was saved every 4 ps.

File Name: Supplementary Movie 3

Description: Simulation on diffusion behavior of p-toluene sulfonic acid (PTSA) in the biphasic system. A 3.7 nm × 3.7 nm × 7.8 nm simulation box was composed of 10 acids molecules, 200 octanoic acid molecules, and 1718 water molecules. Equilibrium MD simulation was run for 20 ns. The initial 5 ns was used to ensure the system reached equilibrium and the data were averaged over the last 15 ns. For each simulation, we used a time step of 2 fs and the trajectory was saved every 4 ps.
